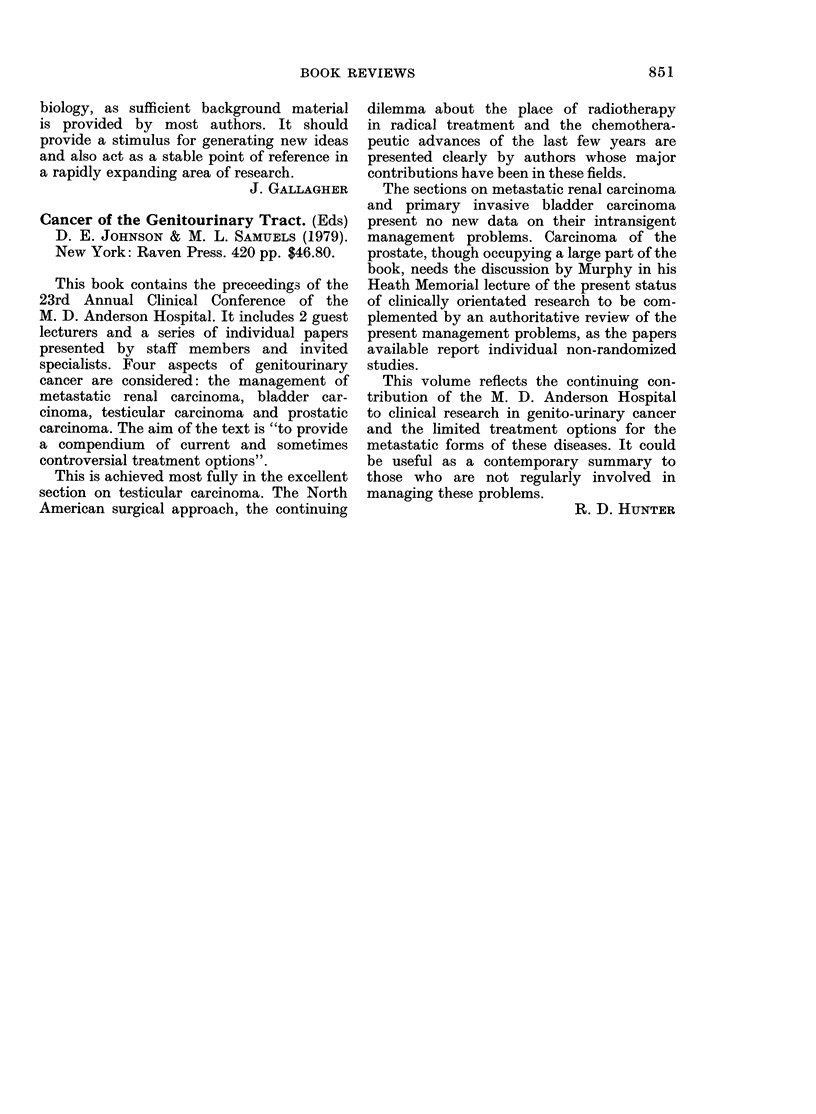# Cancer of the Genitourinary Tract

**Published:** 1980-05

**Authors:** R. D. Hunter


**Cancer of the Genitourinary Tract.** (Eds) D. E. Johnson & M. L. Samuels (1979). New York: Raven Press. 420 pp. $46.80.

This book contains the preceedings of the 23rd Annual Clinical Conference of the M. D. Anderson Hospital. It includes 2 guest lecturers and a series of individual papers presented by staff members and invited specialists. Four aspects of genitourinary cancer are considered: the management of metastatic renal carcinoma, bladder carcinoma, testicular carcinoma and prostatic carcinoma. The aim of the text is "to provide a compendium of current and sometimes controversial treatment options".

This is achieved most fully in the excellent section on testicular carcinoma. The North American surgical approach, the continuing dilemma about the place of radiotherapy in radical treatment and the chemotherapeutic advances of the last few years are presented clearly by authors whose major contributions have been in these fields.

The sections on metastatic renal carcinoma and primary invasive bladder carcinoma present no new data on their intransigent management problems. Carcinoma of the prostate, though occupying a large part of the book, needs the discussion by Murphy in his Heath Memorial lecture of the present status of clinically orientated research to be complemented by an authoritative review of the present management problems, as the papers available report individual non-randomized studies.

This volume reflects the continuing contribution of the M. D. Anderson Hospital to clinical research in genito-urinary cancer and the limited treatment options for the metastatic forms of these diseases. It could be useful as a contemporary summary to those who are not regularly involved in managing these problems.

R. D. Hunter